# Comparative whole transcriptome analysis of gene expression in three canine soft tissue sarcoma types

**DOI:** 10.1371/journal.pone.0273705

**Published:** 2022-09-13

**Authors:** Lydia Lam, Tien Tien, Mark Wildung, Laura White, Rance K. Sellon, Janean L. Fidel, Eric A. Shelden

**Affiliations:** 1 School of Molecular Biosciences, College of Veterinary Medicine, Washington State University, Pullman, WA, United States of America; 2 Veterinary Clinical Sciences, College of Veterinary Medicine, Washington State University, Pullman, WA, United States of America; 3 Washington Animal Disease Diagnostic Laboratory, College of Veterinary Medicine, Washington State University, Pullman, WA, United States of America; Colorado State University, UNITED STATES

## Abstract

Soft tissue sarcomas are pleiotropic tumors of mesenchymal cell origin. These tumors are rare in humans but common in veterinary practice, where they comprise up to 15% of canine skin and subcutaneous cancers. Because they present similar morphologies, primary sites, and growth characteristics, they are treated similarly, generally by surgical resection followed by radiation therapy. Previous studies have examined a variety of genetic changes as potential drivers of tumorigenesis and progression in soft tissue sarcomas as well as their use as markers for soft tissue sarcoma subtypes. However, few studies employing next generation sequencing approaches have been published. Here, we have examined gene expression patterns in canine soft tissue sarcomas using RNA-seq analysis of samples obtained from archived formalin-fixed and paraffin-embedded tumors. We provide a computational framework for using resulting data to categorize tumors, perform cross species comparisons and identify genetic changes associated with tumorigenesis. Functional overrepresentation analysis of differentially expressed genes further implicate both common and tumor-type specific transcription factors as potential mediators of tumorigenesis and aggression. Implications for tumor-type specific therapies are discussed. Our results illustrate the potential utility of this approach for the discovery of new therapeutic approaches to the management of canine soft tissue sarcomas and support the view that both common and tumor-type specific mechanisms drive the development of these tumors.

## 1. Introduction

Canine soft tissue sarcomas (STS) are a large group of neoplasms derived from cells of mesenchymal origin that display similar histological appearance and clinical behavior [1–3]. Soft tissue sarcomas occur frequently in dogs, accounting for up to 15 percent of all skin and subcutaneous tumors. Up to 95,000 dogs are diagnosed with STS in the United States each year [4] and 20 to 30 percent of them die as a result of their disease [2]. In humans, STS are comparatively rare in adults, but they make up nearly 7% of cancers in adolescents [5]. Patients with unresectable, metastatic or advanced disease have a median survival time of 14 months [6]. The most common treatment for both canine and human STS is surgical excision with wide margins followed by radiation or chemotherapy to eliminate microscopic disease [7–9]. However, these pseudoencapsulated tumors often infiltrate extensively along fascial planes with indistinct histologic margins and can often occur on extremities; therefore, complete surgical excision with curative intent is often difficult to achieve. In the dog, recurrence is common, and high-grade sarcomas have at least a 40% chance of metastasizing [10].

Common canine STS histological subtypes include fibrosarcomas, peripheral nerve sheath tumors, and perivascular wall tumors [2]. STS are typically diagnosed by examination of tumor histology using hematoxylin and eosin-stained tissue sections. However, due to their similar histological appearance, differentiation of histological subtypes of STS using these methods is difficult [11, 12]. The diagnosis of specific tumor subtype can have both prognostic and therapeutic value [4, 11–13] and errors may have significant therapeutic consequences [14, 15]. The value of histologic type in canine soft tissue sarcomas is less clear, potentially due to the lack of consistent methods for accurate differentiation of STS type (3). To address this, several previous studies have examined the utility of immunological and genetic markers for canine STS. For example, expression of the S100 family of calcium binding proteins and the intermediate filament proteins desmin and vimentin have been examined as a diagnostic marker for peripheral nerve sheath tumors [16], and the absence of staining for S100, desmin, CD31, and AE1/AE3 has been used to diagnose fibrosarcomas [12]. Klopfleisch et al examined the expression of mRNAs in canine fibrosarcomas and peripheral nerve sheath tumors using microarrays and identified 77 differentially expressed transcripts [17]. However, in a subsequent study only 2 were deemed useful for differentiation of tumor types using reverse transcriptase polymerase chain reaction (RT-PCR) methods [18]. Additionally, some studies have indicated that immunological staining for some markers, such as S100, provides poor diagnostic accuracy [13], and that over 20% of canine and human STS are not accurately diagnosed using these approaches [12].

Immunohistochemistry, microarrays, and RT-PCR are difficult to use for quantitative studies of gene expression and have comparatively low sensitivity. High-throughput nucleotide sequencing methods such as RNA-seq address these limitations and are revolutionizing clinical diagnostic procedures [19]. Applications in veterinary practice are also emerging [20]. RNA-seq can provide quantitative expression data for thousands of genes in a single sample, and computational approaches have been developed to analyze resulting complex data sets [21]. RNA-seq has been used to distinguish tumor subtypes in human sarcoma [22, 23] and canine melanoma, hemangiosarcoma, urothelial carcinoma and osteosarcoma [24–26]. Comparison with gene expression patterns in appropriate normal controls can generate insights into tumor etiology and behavior that can form the basis of therapeutic interventions [27]. To date, however, we are aware of only one study that has examined canine STS using these methods, and tumor type-specific gene expression patterns were not assessed in that study [28].

In the present study, we examined gene expression levels in formalin-fixed, paraffin-embedded canine STS obtained during routine clinical practice using RNA-seq. We established procedures for clustering tumors based on similar gene expression patterns and identified normal canine tissue data sets for analysis of tumor etiology. Our results support and extend previous studies showing the potential of gene expression patterns to identify tumor-subtypes in STS and underscore commonalities between canine and human STS subtypes. Our results also identify potential common and tumor-type specific drivers of tumorigenesis in canine STS and as well as potential therapeutic compounds targeting them that may have clinical value in the treatment of associated disease.

## 2. Materials and methods

### 2.1 Animals and samples

Sixteen samples were obtained from formalin-fixed, paraffin-embedded (FFPE) canine soft tissue sarcomas that were retroactively identified from archived tumor tissue submitted to the Washington Animal Disease Diagnostic Laboratory (WADDL) from the Washington State University Veterinary Teaching Hospital between 2015 to 2019. Samples were selected based on 1) adequate tissue volume for sampling, and 2) having at least one year of follow-up available. Clinical follow-up data were obtained in a retrospective manner through medical records and phone contact with the referring veterinarian and owners. The sectioned and H&E-stained slides from the samples were evaluated by a single board-certified anatomic pathologist (LW) for histologic grade (including mitotic index, percentage of necrosis, and differentiation score) (3), presence of inflammation, tumor subtype, and surgical margin status. Clinical data can be found in S1 Table. All work with canine tissue samples was approved by Washington State University’s Institutional Animal Use and Care (IACUC) committee.

### 2.2 RNA extraction, preparation, and sequencing

Two 50-micron thick sections were obtained from each FFPE tumor sample and processed for total RNA extraction using an Qiagen RNeasy FFPE kit. One microgram of total RNA was enriched for mRNA using Ribominus Eukaryotic V2 (Invitrogen). Sequencing libraries were constructed using 200 nanograms of enriched mRNA and the Ion Total RNA-Seq Kit V2 (Life Technologies) without further fragmentation while all purification and size selection were performed using AMPureXP beads (Beckman Coulter genomics). Emulsion PCR to generate library decorated ion spheres was performed using an Ion Chef (Life). Libraries were sequenced in two groups with an Ion Proton and six Ion P1 semiconductor sequencing chips, using Ion P1 Hi-Q reagents (Life). RNA seq reads were aligned to a recent canine reference genome (ROS-canfam-01) using STAR2 [29] and counted using htseq-count [30]. FASTQ files and counts of aligned reads are available from the NCBI GEO repository, accession number GSE208664. An initial analysis of tumor specific genes was conducted after batch effects adjustment using the R package “Combat-seq” [31], however we observed no subsequent improvement in the number of identified tumor specific differentially expressed genes (DEGs) and this approach was not used for further analysis.

### 2.3 Additional RNA-seq data

RNA-Seq datasets obtained from normal canine tissues were downloaded from the NCBI sequence read archive database (SRA) using “prefetch”, and FASTQ files were produced using “fastq-dump”. Normal canine endothelial and vascular smooth muscle cell [32], soft tissue [33], skin [34], and stromal tissue [35] data sets were obtained from SRA project archives PRJNA484120, PRJNA449364, PRJNA516470, and PRJNA557680, respectively. RNA-seq data for human soft tissue sarcomas was downloaded from The Cancer Genome Atlas Program (TCGA) using the R package “TCGABiolinks” [36].

### 2.4 Data analysis

Principle component analysis of tumor data was conducted using the R package “FactoMineR” [37]. Comparisons of gene expression levels between tumor types and between tumor and normal tissue datasets was conducted using “DESeq2” [38]. Differentially expressed genes (DEGs) were defined by an adjusted p value (padj) of less than 0.05 and a log2fold change greater than 1. Identified DEGs were examined for statistically significant enrichment of genes expressed by human soft tissue sarcomas (HP:0030448, hpo.jax.org) using the “enrichr” function in the R package “clusterprofileR” [39]. DEGs with outlying expression levels in each tumor group were identified using the R base function “boxplot” using default parameters, and the number of such DEGs for each sample is provided in S3 Table. Comparative analysis of gene expression patterns between canine and human soft tissue sarcomas was conducted using the R package “AICcmodavg” [40] to calculate the Akaike’s information criterion (AIC). Gene set overrepresentation analysis was conducted by querying the ENRICHR web site [41] using the R package “enrichR” [42] and data libraries listed in S8 Table. Unsupervised hierarchical clustering and generation of heatmaps was conducted using the “heatmap.2” function in the R package “gplots” [43]. Custom R scripts were written to perform permutation tests for statistical significance and to identify and present the most significant gene set enrichments. Dot-plots were produced using the R package “ggplot2” [44]. All scripts can be obtained from the GitHub repository “eshelden/cSTS” or by request.

## 3. Results

### 3.1 Canine soft tissue sarcoma tumor types can be differentiated by gene expression patterns

**Fig 1:**
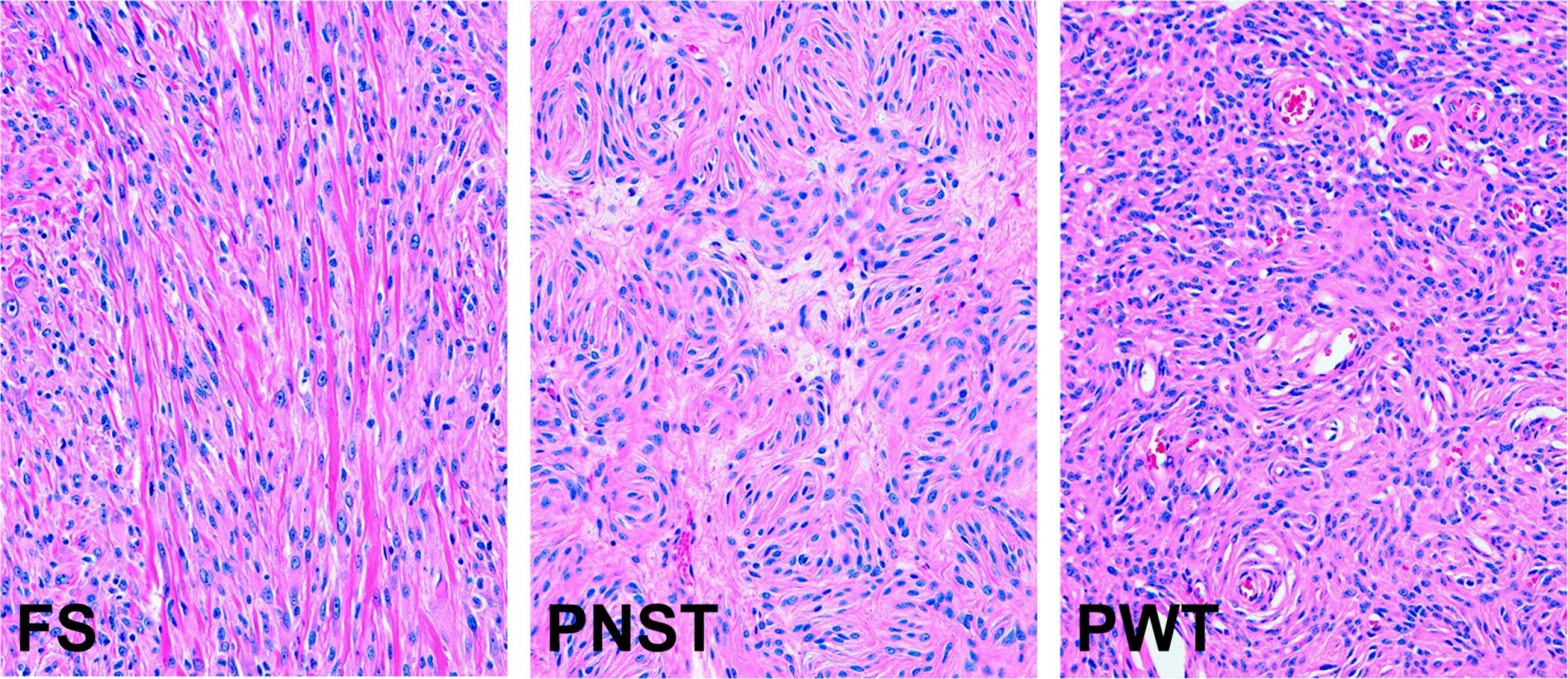
Typical histologies of canine soft tissue sarcomas. Hematoxylin and eosin-stained paraffin sections of canine soft tissue sarcoma tumors, showing typical morphologies of fibrosarcomas (FS), peripheral nerve sheath tumors (PNST) and perivascular wall tumors (PWT).

Tumors were first placed into groups (fibrosarcomas (FS), peripheral nerve sheath tumors (PNST) and perivascular wall tumors (PWT)) based on histological analysis. Initial tumor designations and characteristics can be found in S1 Table. Fibrosarcomas were characterized by the presence of long cellular streams containing collagenous stroma. Peripheral nerve sheath tumors displayed short, interlacing fascicles often with storiform pattern (fingerprint) and nuclear palisading. Perivascular wall tumors displayed perivascular patterns often including perivascular whorls. Typical examples are shown in Fig 1.

An initial unsupervised clustering of samples using principal component analysis of the 500 most variably expressed genes showed clustering of most samples by assigned tumor type, but some clustered with other tumor types (A in S2 Fig). K-means clustering also failed to place samples into groups corresponding to histological diagnoses (B in S2 Fig). Differential expression analysis of genes comparing sample groups based on histology identified 761 DEGs specific for fibrosarcomas, 233 DEGs specific for perivascular wall tumors, and 11 DEGs specific for peripheral nerve sheath tumors. A permutation test revealed that the number of DEGs identified for fibrosarcomas and perivascular wall tumors, but not peripheral nerve sheath tumors were significantly greater than expected from random sampling (p < .01, p < .05 and p > .05, respectively). However, counting the number of DEGs for each sample where its expression level was an outlier among samples of the same histological subtype identified two (S1-10 and S2-8), that poorly matched their assigned subtype (S3 Table). We first removed these samples and recalculated tumor-type specific DEGs, then performed unsupervised hierarchical clustering of all tumors using the 185 most variable resulting DEGs for each tumor type. This analysis reassigned the two outliers (S1 Table). The final number of DEGs calculated for each tumor group was 2090 (FS), 875 (PWT) and 291 (PNST). Permutation tests showed that reassignment increased the significance of resulting DEG identification from p = .003 to p < .001 (FS), p = .027 to p = .002 (PWT), and p = .297 to p = .019 (PNST). The final list of DEGs specific to each tumor type is provided in S5 Table.

**Fig 2:**
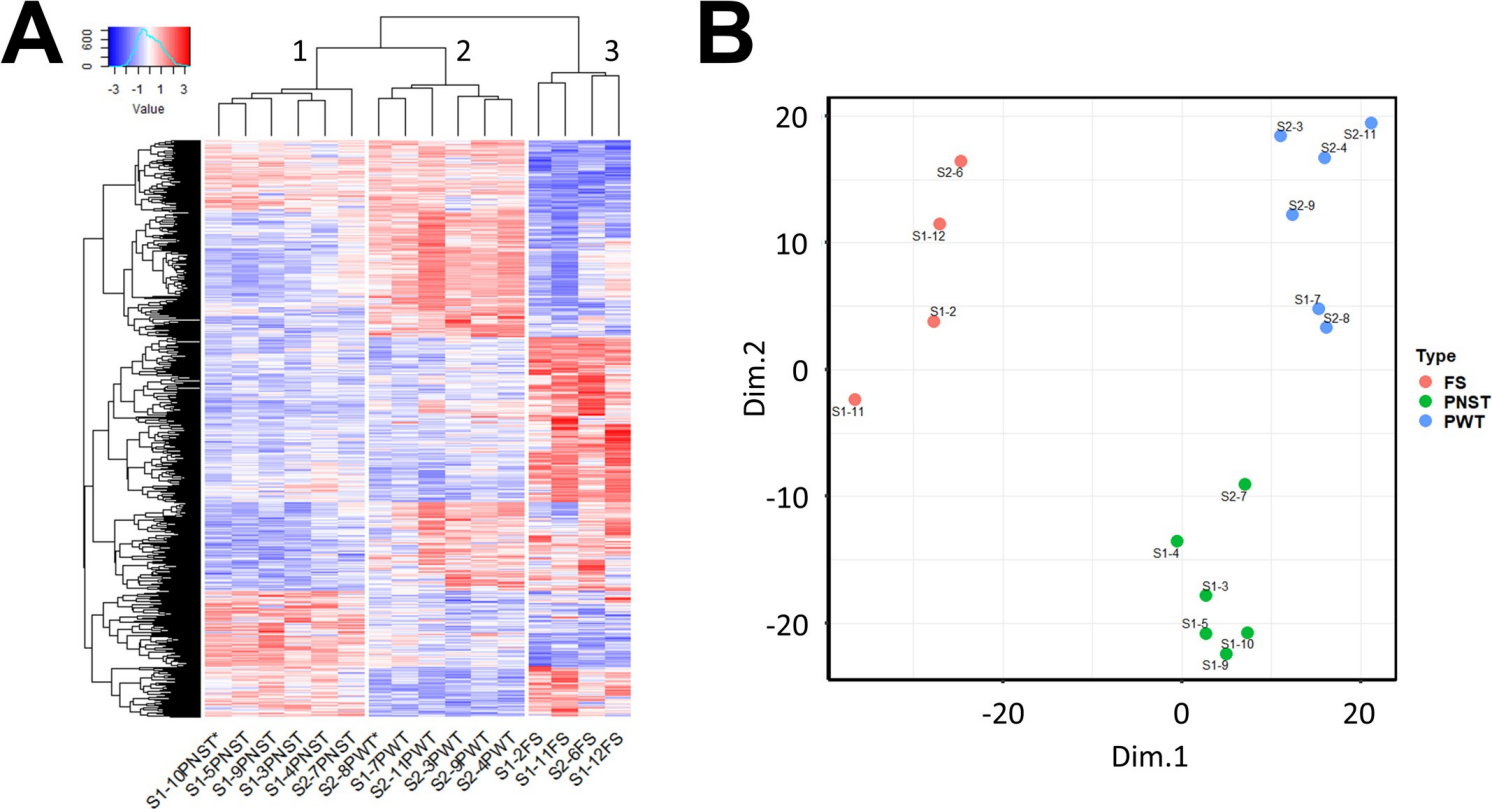
Clustering of canine soft tissue sarcoma tumor samples using gene expression patterns. A) A heat map showing upregulated genes in red and downregulated genes in blue of 2387 tumor-type specific genes. Dendrograms show results of unsupervised hierarchical clustering of samples and genes. B) Principal component analysis of 16 canine soft tissue sarcomas using the 250 most significantly different genes associated with each tumor type. Values are z-scores of expression for each gene. Asterisks indicate reassigned samples.

Hierarchical clustering of all samples using normalized expression of final DEGs is shown in Fig 2A. To avoid biasing this analysis for DEGs identified in fibrosarcomas, only the most significantly different 291 DEGs for each tumor type were included. Examination of the resulting figure suggests that peripheral nerve sheath tumors and perivascular wall tumors (groups labeled 1 and 2) are genetically more like each other and have less within-subtype variation in gene expression than fibrosarcomas (group 3). In contrast, fibrosarcomas are readily distinguishable from other tumor types, but also display a high degree of within-subtype variability, especially for upregulated genes. Unsupervised principal component analysis of tumor samples based on the 250 most significant DEGs for each tumor type unambiguously distinguishes three tumor groups (Fig 2B). Perivascular wall and peripheral nerve sheath tumors group together on principle component axis 1 (Dim. 1) which explains 42.2% of total variance, while fibrosarcoma tumor samples are located further from the other two tumor types along Dim.1 as well as each other on Dim.2, which explains 29.6% of total variance.

The 25 genes with the most significantly higher or lower expression in specific tumor subtypes are presented in Table 1 along with a classification of their major function. Graphs of normalized expression values for these genes are provided in S3–S5 Figs. Consistent with the heatmap of gene expression shown in Fig 2A, fibrosarcomas showed the greatest differences in expression of transcription factors, relative to the other two tumor types. Expression of the only growth factor coding gene in the top 25 most significant DEGs, *GDF11*, was also associated with fibrosarcomas. In contrast, PNST tumors expressed relatively low numbers of genes associated with mitosis and processes such as protein synthesis. Interestingly, PWTs expressed a comparatively large number of DEGs associated with apoptosis. We also examined expression of genes for their potential to serve as tumor type-specific markers. We identified 10 DEGs (*CACNA2D1*, *CDK2AP1*, *GALNT5*, *GNAS*, *HOXA9*, *MAST2*, *PLXNA4*, *POU6F1*, *SALL1*, *SMAD9*) whose normalized expression levels in all fibrosarcoma samples was at least two-fold greater than that in all samples for both other tumor types. No individual DEGs expressed at higher levels in PNSTs or PWTs met this criterion. However, we identified 10 pairs of annotated DEGs expressed by PNSTs (*ATP1A3* and *UNC79*; *BMX* and *GRIN1*, *PTPRN2*, *SEZ6L2*, or *XKR7*; *NRXN1* and *GRIN1*, *KCNH2*, *PTPRN2*, *SEZ6L2*, or *XKR7*), and 5 pairs of annotated and putative genes expressed by PWTs (*FAM177B* and *LOC102152746*, *LOC119873485*, or *LOC119874346*; *NEGR1* and *LOC119869901*; *PRND* and *LOC119873485*) where one or the other gene displayed a normalized expression level at least 1.8-fold greater than that for all samples in both other tumor types.

**Table 1:** Individual tumor type’s top 25 genes with the most significantly higher or lower expression. Genes listed are increased (up) and decreased (down) differentially expressed genes (DEGs) identified using DESeq to compare expression levels of genes in one tumor type against that for both other tumor types. All DEGs were identified using a minimum significance of padj < .05 and a fold change of greater than 2. The 25 genes in each group with the greatest significance determined using adjusted p values are shown. Genes listed in bold were cross referenced with the term “cancer” in at least 50 published manuscripts.

	FS up	FS down	PNST up	PNST down	PWT up	PWT down
Apoptosis			XKR7	SRGN	PAWR, SAMD8, UBXN2A	
Biosynthesis			CERS4	**RRM2**		
Cell adhesion	**CADM1**	DNAH3, TNR	NRXN2		LRRTM2	
Cell Cycle Regulation	CDK2AP1			**CCNA2, CDK1**		
Cell Stress, Hypoxia			TMEM145			
Cell-cell signaling	NDNF					
Cytoskeleton	MAST2, MTCL1	DNAH3	WRAP73	DIAPH3		
Extracellular Matrix	ADAMTS7	TNXB, ITIH5	FBLN2			CHPF2, FBN2, LAMC3, MXRA5, TNN
Growth Factors	GDF11					
Immune Responses		CLEC3B	DLA88		ELMOD2, **IFNAR1**	IL16
Ion Transport		KCNQ2	AHNAK2, UNC79, ATP1A3		ATP13A3	
Metabolism	ALDH1L2		FOXRED2		**FBP1, PDK4**	**G6PD**, NDUFA13
Mitosis				KIF11, KIF14, KNL1, SHCBP1, SMC2	CEP57L1, PARD6B	
Other		RIC3		C31H21orf91	GIN1, NHLRC2	
Protein synthesis, modification	GALNT5		WFDC3	**ATAD2, SACS,** UBE2D1, TRNAK-CUU-12, TRNAM-CAU-5, TRNAV-AAC-5, TRNAV-CAC, TRNAV-UAC-3	FKTN, UBE2B, TRNAE-UUC, FYTTD1	CLPP, FKBP10, LARGE1, RRBP1, **PP1A**
Receptors and their regulation	UNC5C, **ROBO1**, S1PR3, PLXNA4, CRLF1	SEZ6L2	SEZ6L2, CHRNB2		CALCRL	AGAP3, **EPHB3**
Signal Transduction	**MAPKAPK2**	BMPER, ABLIM1, EPS15, MAPK8IP2, HHIP, FGD6, MAPK11, **DEPTOR**, RALGPS1	**PTPRN2**, RUNDC3A, MAST1, MAPK4	GNG2, **KRAS**, TRIM59	**PTPN2, RAP1A,** CRYBG3	ADAP1, NCLN, TMEM119
Transcription factors and regulation	SALL1, POU6F1, **TRIM24**, SMAD9, **HOXA9**, CPHXL, ZBTB47, OLFML2A	PEG3, RXRG, ELAVL3, BBX, ZMAT1	ZBTB12	**REST**	MIR195	**NCOR2**, ZBTB47
Vesicles, Membrane Transport	DYSF, STXBP6	ABCB11, ABCA9, CAVIN2	SLC46A1, ABCA2, ATP13A2, SLC22A17, SLC1A6, MFSD3	RASSF9	**TTN**, RAB8B, DENND6A	ANXA8L1, ASPSCR1, EPN1, SLC1A4, SURF4

All genes in Table 1 showed at least a two-fold increase or decrease in average expression value relative to the other tumor types and expression differences were significant at a level of at least padj < 0.05. For genes whose average expression was higher (“up” in Table 1) in each tumor subtype, the lowest and average log-normalized expression values were 3.06 (HOXA9) and 5.84 for fibrosarcomas, 1.6 (XKR7) and 5.33 for peripheral nerve sheath tumors, and 3.33 (FBP1) and 6.83 for perivascular wall tumors. For comparison, the average log normalized expression value for the transcription factor SNAIL (SNAI1), which play a key role regulating the behavior of some sarcoma cell types [45, 46] was 3.50 for all samples in this study, while that of keratin 13 (KRT13), which is expressed in epithelial cell types [47] but is not expected to be significantly expressed in soft tissue sarcomas, was -1.47. For genes expressed at lower levels in each tumor subtype (labeled “down” in Table 1), the average log normalized expression value of genes in the other two tumor subtypes for the 25 down-regulated genes was 5.27 for fibrosarcomas, 5.22 for peripheral nerve sheath tumors and 6.5 for perivascular wall tumors. Log normalized expression values for all genes in all samples can be found in S2 Table, and values for statistical significance and fold change for all tumor type specific DEGs can be found in S5 Table. Taken together, the values described above suggest that the genes listed in Table 1 represent both statistically and biologically significant differences among tumor subtypes.

**Fig 3:**
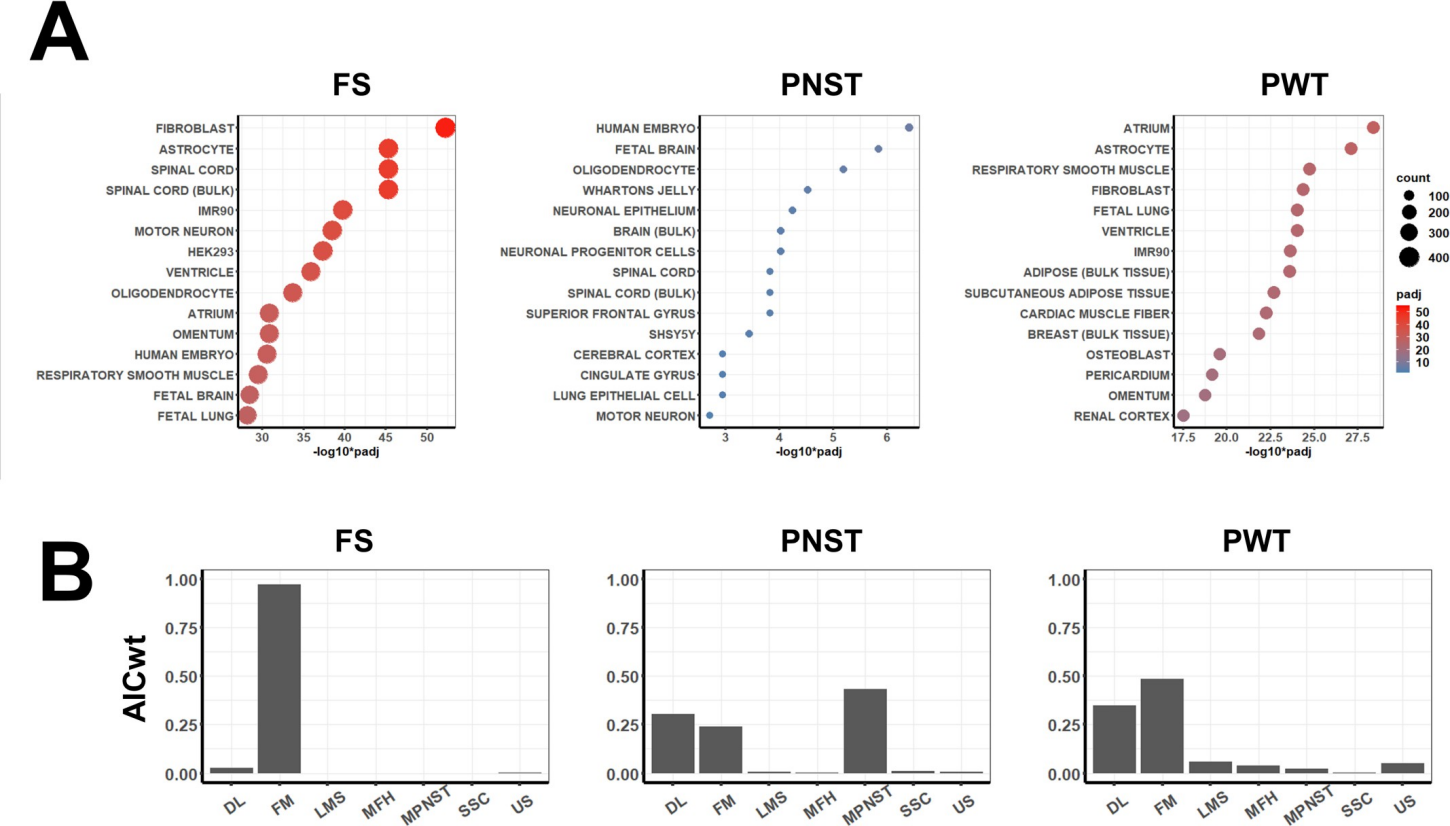
Comparative gene expression analysis of canine soft tissue sarcomas and human cells, tissues and tumors. A) Dot plots showing results of gene set over-representation analysis of canine soft tissue sarcoma tumor-type specific DEGs using databases for human tissues and cell lines. B) Plots of Akaike weights obtained using normalized gene expression levels of canine soft tissue sarcoma tumor-type specific DEGs and the same genes expressed in various human soft tissue sarcomas obtained from the NCBI Genomic Data Commons. Abbreviations are: DL: dedifferentiated liposarcomas, FM: fibromyxosarcoma, LMS: leiomyosarcoma, MFH: undifferentiated pleomorphic sarcoma, MPNST: malignant peripheral nerve sheath tumors, SSC: synovial sarcoma, US: undifferentiated pleomorphic sarcoma.

Fig 3A shows gene set over-representation analysis of tumor type specific genes using databases for genes characterizing human tissues and cell lines. Names of genes enriched for each group, ordered by log2 fold-change, are provided in S7 Table. Genes characterizing canine fibrosarcomas (FS) showed the most significant enrichment for gene sets defining human fibroblasts; genes expressed by the human IMR90 lung fibroblast cell line were also enriched. Genes characterizing canine peripheral nerve sheath tumors (PNST) were most significantly enriched for gene sets expressed by neuronal tissues including fetal brain, oligodendrocytes and human SHSY5Y neuroblastoma cells. Perivascular wall tumors (PWT) expressed genes significantly enriched for genes expressed by vascular tissues (atrium, ventricle) as well as respiratory smooth muscle tissues and fibroblasts. Analysis of the correlation between the expression levels of canine tumor-type specific genes and their expression in human soft tissue sarcomas are shown in Fig 3B. Expression patterns of canine fibrosarcoma DEGs were most like that of human fibromyxosarcomas, and this similarity was significantly greater than the similarity to all other tumor types. Similarly, expression patterns of canine peripheral nerve sheath tumors showed the highest correlation with human malignant peripheral nerve sheath tumors. Expression of genes specific for canine perivascular wall tumors showed significantly greater similarity to both human fibromyxosarcomas and dedifferentiated liposarcomas than to other human soft tissue sarcoma types.

### 3.2 Tumor type specific gene expression patterns associated with tumorigeneses

Normal tissues providing the greatest statistical enrichment in genes associated with human soft tissue sarcomas were identified for each tumor type (Methods and S4 Table) and used to identify tumorigenesis associated DEGs (see S6 Table). These comparisons identified 2144 upregulated and 1658 downregulated genes for fibrosarcomas, 1903 upregulated and 1844 downregulated genes for peripheral nerve sheath tumors and 2528 upregulated and 1914 downregulated genes for perivascular wall tumors. Additionally, 613 upregulated and 270 down-regulated genes were shared by all three tumor types. Upregulated genes comprised similar fractions of tumor type specific DEGs for each tumor type (FS: 56.4%; PNST: 50.78%, PWT: 56.9%) suggesting that the selection of normal tissues gene expression data for comparison were similarly appropriate.

**Fig 4:**
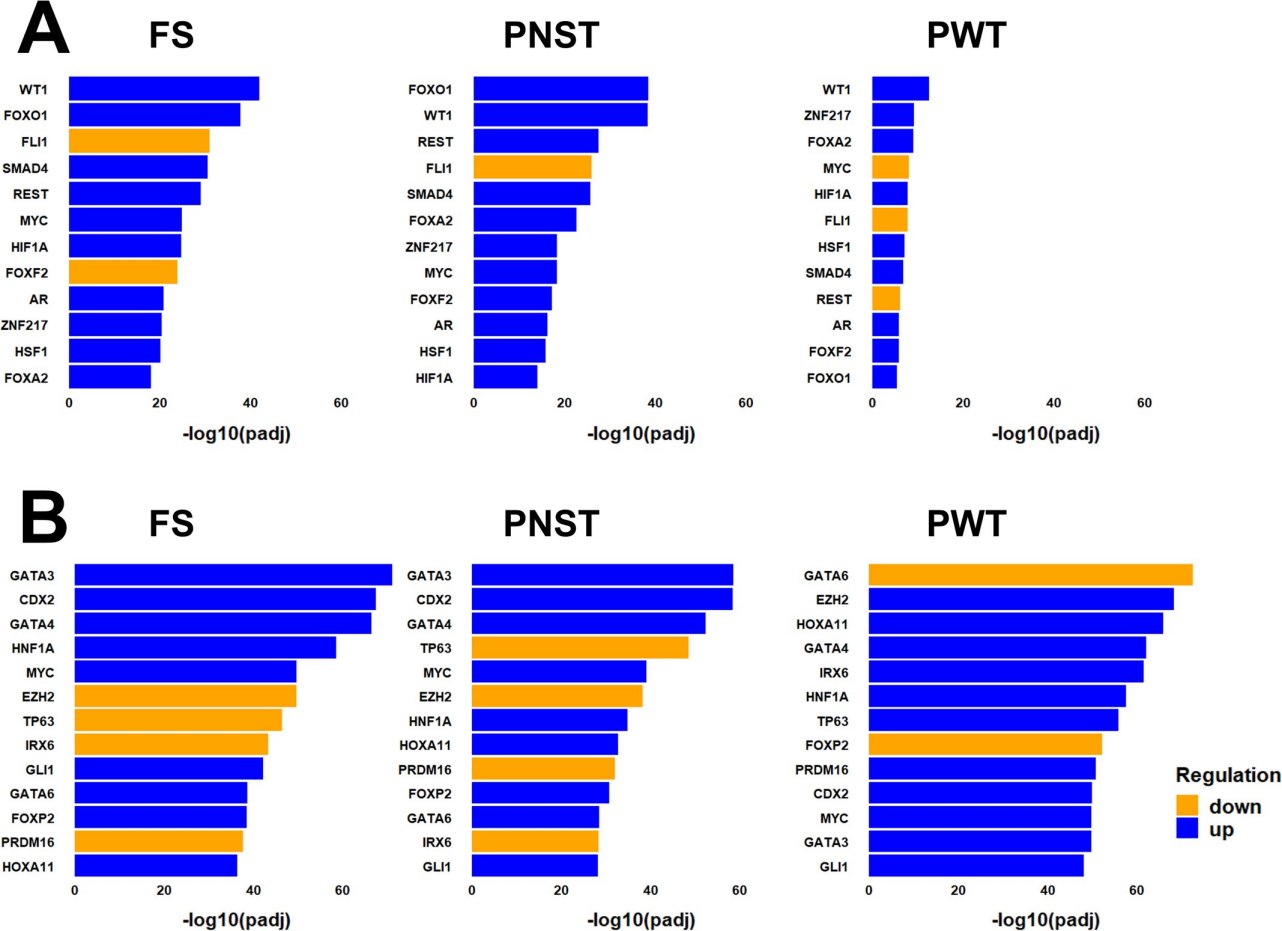
Common transcription factors associated with gene alterations in canine soft tissue sarcomas compared to normal tissues. The top 12 most significant common transcription factors identified from a gene set enrichment analysis using lists of tumor-type specific up (A) and down (B) regulated DEGs is shown for each tumor type. Lengths of bars represent the statistical significance of each association, and colors indicate if the results imply upregulation (blue) or downregulation (orange) of the activity of each transcription factor.

**Fig 5:**
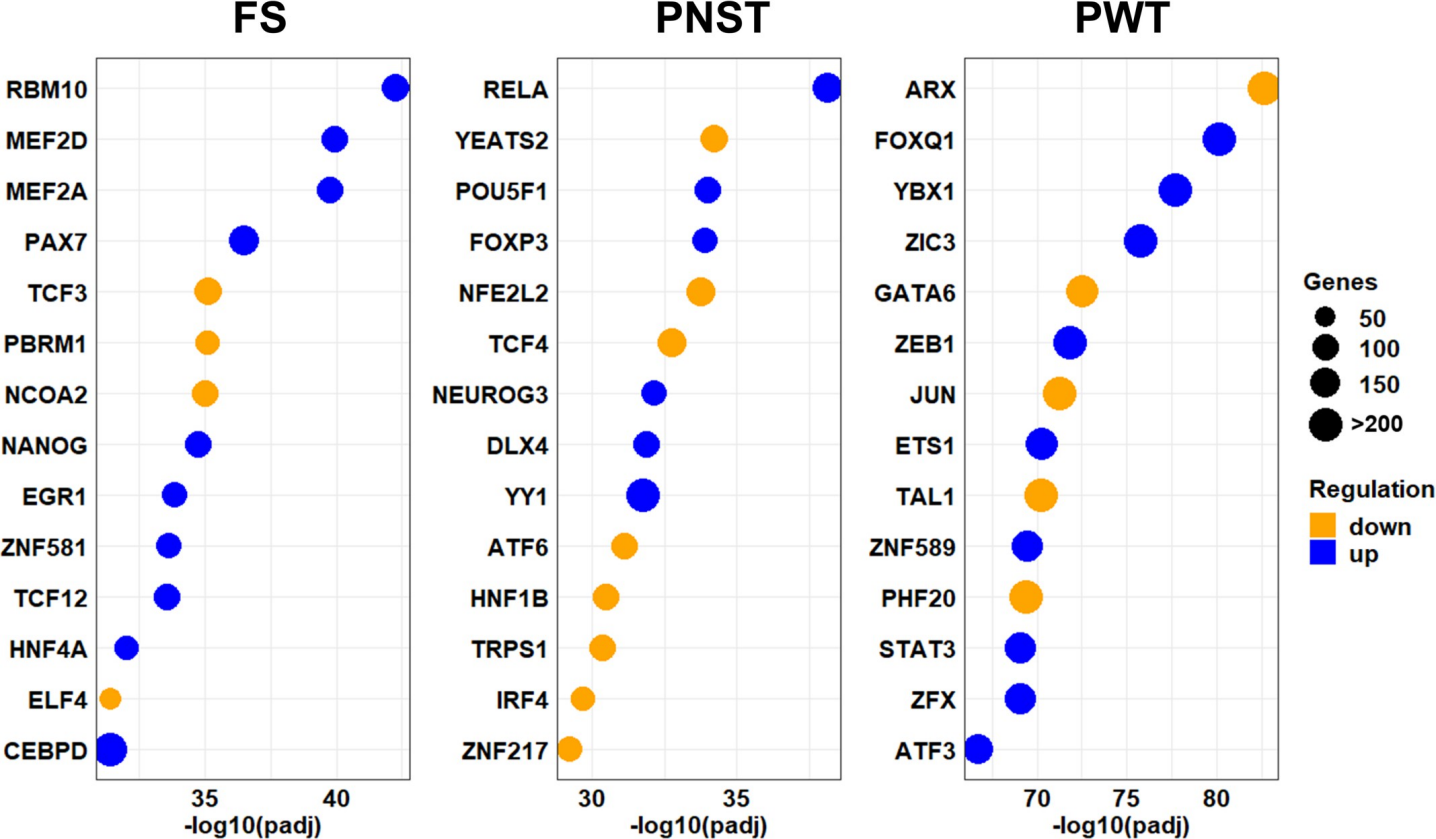
Tumor-type specific transcription factors associated with gene alterations in canine soft tissue sarcomas compared to normal tissues. The top 14 most significant tumor-type specific transcription factors identified from a gene set enrichment analysis using lists of tumor-type specific up (A) and down (B) regulated DEGs. Values for “Genes” and dot sizes represent the number of DEGs matched to each transcription factor listed.

Next, we identified common (Fig 4) and tumor type-specific (Fig 5) transcription factors associated with these DEGs using gene set enrichment analysis. Terms, p-values, and associated genes ordered by log2fold change in expression levels are provided in S7 Table. Results shown in Fig 4 were produced by conducting gene set enrichment analysis (GSEA) using all upregulated (Fig 4A) or downregulated (Fig 4B) DEGs identified for each tumor type, of which 1359 DEGs were common to all three tumor types. The 12 most significantly enriched gene sets associated with transcription factor activity are shown for each analysis. Note that for some transcription factors, such as FLI1, upregulated expression of a group of genes is known to be associated with a decrease in activity of that transcription factor. Bars representing these transcription factors are colored orange in Fig 4A. Similarly, decreases in expression of some genes is associated with increased activity of a transcription factor, and bars representing such transcription factors are colored blue in Fig 4B. Examination of Fig 4 reveals that activation of the MYC proto-oncogene is significantly associated with the downregulated DEGs identified for all tumor subtypes in this study (Fig 4B) as well as the upregulated DEGs for fibrosarcomas and peripheral nerve sheath tumors (Fig 4A). Upregulated DEGs specific to perivascular wall tumors were enriched in genes associated with MYC downregulation (Fig 4A). However, the significance of this association was much lower than that for MYC upregulation for these tumors shown in Fig 4B as well as all other groups of DEGs. Taken together, results shown in Fig 4 support the view that upregulation of MYC transcription factor activity is common to all three tumor subtypes in this study. Other well-known transcription factors whose function positively associated with upregulated DEGs in all tumors (Fig 4A) include the proto-oncogenes WT1 and ZNF217, and enhancers of cell stress responses (HSF1, HIF1). Similarly, results obtained using downregulated DEGs (Fig 4B) include transcription factors previously associated with poor prognosis for human soft tissue sarcomas (GATA4, GATA3).

To identify molecular mechanisms that might be involved in the pathogenesis or etiology of individual tumor types, we conducted gene set enrichment analysis using DEGs specific to individual tumor types. Fig 5 shows the 14 most significantly enriched tumor type-specific transcription factors identified by conducting gene set enrichment analysis using only the up- and down- regulated tumorigenesis-associated DEGs specific to each tumor type. Transcription factors whose activation was most significantly associated with fibrosarcomas include *RBM10*, *MEF2D*, *MEF2A*, and *PAX7*. Decreased activities of tumor suppressors *TCF3* and *PBRM1* were also among the most significant associations. Similarly, positive activation of transcription factors *FOXP3*, *RELA*, and *POU5F1* was associated with PNSTs and have been previously associated with tumorigenesis in other cancers [48–50]. In contrast, *NFE2L2* downregulation was significantly associated with DEGs expressed by PNSTs, consistent with its reported function as a tumor suppressor [51]. Finally, DEGs expressed by PWTs were significantly enriched for transcription factors associated with tumor growth (*YBX1*) and metastasis (*FOXQ1*) as well as decreased activity of the development/differentiation associated transcription factor *ARX*.

## 4. Discussion

The present study examined tumor-type specific gene expression patterns in canine soft tissue sarcomas using RNA-seq of formalin-fixed, paraffin-embedded (FFPE) samples. Although the sample size used in this study was small, the number of samples for each tumor type was consistent with that used in several previous studies [17, 28, 52–54]. Importantly, our sample size was sufficient to identify 3256 differentially expressed genes among these three distinct tumor types with high statistical significance. Formalin-fixed, paraffin-embedded specimens are widely available and can be a valuable source of samples for analysis. However, extraction and analysis of RNA from archived specimens can be difficult and result in recovery of degraded RNAs that are poorly suited for analysis using RT-PCR or qRT-PCR [55, 56]. Indeed, despite considerable effort, we were unable to reliably conduct RT-PCR validation of differentially expressed genes identified in this study. However, since RNA-seq generates nucleotide sequence data from fragmented cDNAs, it is more tolerant of RNA degradation than other methods used for identifying RNA species, and many studies have shown that results obtained from RNA-seq analysis of formalin-fixed specimens are comparable to those obtained using fresh frozen specimens [57, 58]. An additional issue is that clinical specimens, such as those used in the present study, are frequently collected without matched control normal tissues. Here, we addressed this issue by conducting a comprehensive comparison of data derived from our tumors with publicly available data from RNA-seq studies of normal canine tissues. These factors suggest that our results should be viewed with some caution and studies with larger sample sizes examining specimens obtained using other methods may be warranted. However, as discussed below, the results obtained in the present study are well supported by results obtained using other methods.

The identification of specific STS subtypes can have prognostic and clinical significance in the management of associated disease in both humans and companion animals [4, 11–13]. Several previous studies have examined immunological and genetic markers for their potential as markers for STS subtypes. For example, expression of NGFR and Olig2 can distinguish PNSTs from PWTs [16, 59]. However, there are controversies and inconsistencies when comparing results of previous studies using different methods. For example, S100 expression has been used to identify PNSTs in many studies, but others have presented strong arguments against its diagnostic value [13]. Thus, additional studies are warranted. Results of the present study are, to our knowledge, the first to examine gene expression differences between different types of canine soft tissue sarcomas using RNA-seq. Our study is most comparable to a previous comparative analysis of gene expression in fibrosarcomas and peripheral nerve sheath tumors using microarrays [17]. In that study, expression of 20 genes was statistically greater in fibrosarcomas, of which 11 (*FHL2*, *FNBP1L*, *PLAGL1*, *FAM110B*, *SLC38A1*, *GNAS*, *HK1*, *BAZ1A*, *BAG2*, *PACSIN3*, *VPS54*) were found to be significantly more highly expressed in FS than either PSNT or PWT in the present study. An additional four previously identified genes (*RAPGEF2*, *SLC20A1*, *CO5A*, *TIGD2*) were also more highly expressed in fibrosarcomas, but the comparison did not exceed the cutoff for significance used in the present study. Similarly, Klopfleisch et al identified 25 genes more highly expressed in PNST than FS, of which 7 (*CLEC3B*, *ABI3BP*, *FMN2*, *PAPLN*, *NUDT2*, *NMUR2*, *TANGO2*) were also expressed at significantly lower levels in FS than either PNST or PWT in the present study. Indeed, *CLEC3B* was among the top 25 most significantly downregulated genes in FS tumors in our study (Table 1, S5 Table). An additional 12 genes (*KIF1B*, *GLI1*, *ROBO2*, *GRAMD4*, *AWGPTL4*, *DOK4*, *TADA2*, *HMG20B*, *SGF29*, *FBX08*, *HNRNPA1*, *EEF1B2*) were expressed at lower levels in FS samples than PNST, but our analysis did not detect differences that were greater than our statistical cutoff. It should be noted that our analysis compared each tumor type against samples in both other tumor types collectively, which may account for some of the differences between our results and those reported previously. None the less, our results confirm differential gene expression for 33 of the previously reported 45 genes differentiating FS from PNST tumors [17]. Wei et al compared gene expression levels in a cross-species comparison of feline, human and canine soft tissue sarcomas with those in non-tumor tissues using RNA-seq analysis [28]. Of the 53 upregulated genes reported, our analysis identified 31 as upregulated in at least one tumor type compared to non-tumor tissues (*ASAP1*, *BICC1*, *C3AR1*, *CD14*, *CD53*, *CD86*, *COCH*, *COL6A3*, *CSF1R*, *CTSK*, *CTSS*, *CYBB*, *DMXL2*, *FAP*, *FBN2*, *FN1*, *GPC3*, *IFI44*, *ITGA4*, *KRT7*, *LAMA4*, *LAPTM5*, *LRRC15*, *MAP1B*, *PTH1R*, *RAB31*, *RGS1*, *SPP1*, *SRGN*, *TGOLN2*, *TLR2*). However, only 8 downregulated genes (*AQP3*, *KRT16*, *PACSIN3*, *PTPN3*, *SERPINB13*, *SMPD3*, *SNTB1*, *TP63*) of the 38 downregulated genes reported by Wei et al, were also identified in our analyses. Additionally, our analysis indicates that many of the most highly downregulated genes in PNST compared to other tumor types are involved in cell cycle progression, mitosis, and cell growth (Table 1). These observations are consistent with previous work showing that PNSTs are comparatively slow growing tumors [60, 61]. Together, these results demonstrate that RNA-seq analysis of FFPE samples using RNA-seq can generate results that are consistent with the results of previous studies using other methods.

In addition to confirming results of previous studies, our results identify potential new candidate genes whose expression could identify soft tissue sarcoma tumor types as well as promote an understanding of their biological differences. For example, fibrosarcomas can be highly aggressive tumors [62]. The matrix metallopeptidase ADAMTS7 expressed in FS was the only member of this gene family found among the top 25 most significantly upregulated genes in STS examined in this study (Table 1), and this family of proteins is known for their promotion of tumor cell invasion and metastasis [63]. Similarly, the only gene among the 25 most significantly upregulated genes encoding a growth factor, GDF11, an atypical member of the TGF-beta superfamily, is also expressed in FS (Table 1). Fibrosarcomas also exhibited the greatest number of transcription factors among the top 25 most significantly upregulated genes (Table 1), including *TRIM24* which acts as an oncogene when overexpressed in several human cancer types [64]. In contrast, as noted above, PNSTs displayed relatively large numbers of downregulated genes involved in regulating cell cycle progression, mitosis, and cell growth, including KRAS. In humans, related malignant peripheral nerve sheath tumors (MPNSTS) show low sensitivity to radiation and chemotherapy [65], which primarily target proliferating cells. Additionally, PNSTs in the present study showed the highest number of upregulated genes involved in transport mechanisms, including ABCA2, a member of the ABC family of membrane transport proteins previously associated with multidrug resistance in some cancers [66]. Perivascular wall tumors have been described as relatively benign STS with a low propensity to metastasize [67]. Consistent with this, the top 25 most significantly under-expressed genes in PWTs included the largest number of genes involved in extracellular matrix regulation. Perivascular wall tumors also had the greatest number of significantly upregulated genes involved in the promotion of apoptotic cell death (Table 1).

Results of the present study may improve our understanding of mechanisms driving the etiology and behavior of canine soft tissue sarcomas and highlight commonalities and differences with other cancer types. Additionally, our results identify potential understudied and tumor-type specific mechanisms that may form the basis of further investigation. For example, our analysis of DEGs between tumors and normal tissues identified gene sets associated with the activation of proto-oncogenes *MYC*, *ZNF217*, and *WT1* (Fig 4). All three are known to play roles in tumorigenesis in a wide variety of cancer types, including soft tissue sarcomas [68–70]. Similarly, our analysis identified upregulation of genes associated with activation of the hypoxia inducible transcription factor, HIF1, in all three tumor types; HIF1 is a known contributor to cancer cell survival in soft tissue sarcomas [71]. It is notable that results obtained for PWTs differed from results obtained for FS and PNST more than the latter two types differed from each other. Of the 23 unique transcription factors shown in Fig 4, gene set enrichment analysis indicated that FS and PNST tumors shared increases or decreases in the function of 22. In contrast, only 14 of 23 transcription factors in PWTs showed the same changes as those indicated in FS and PSNTs. These differences may reflect technical issues with the choice of normal tissue datasets used to identify tumor-type specific DEGs. However, PWTs are heterogenous tumors that arise from several distinct cell types. It is therefore possible that our results are consistent with this heterogeneity and that larger studies with more samples will uncover further molecular distinctions of PWT subtypes [13].

Other results of the present study may highlight differences between soft tissue sarcomas and other cancer types. For example, activation of transcription factors GATA4 and GATA3 was associated with DEGs identified in the present study (Fig 4A). These transcription factors act as tumor suppressors in some cancers [72–75]. However, consistent with results of the present study, their activities promote aggressive behavior in other cancers, including those of mesenchymal origin [76–79]. Our results are therefore consistent with those reported in many previous studies, most of which have been conducted on human tumors, and underscore commonalities in mechanisms responsible for tumorigenesis and cancer behavior in both species. Additionally, results of the present study suggest that previously understudied or novel gene regulatory mechanisms may play roles in canine, and possibly human soft tissue sarcomas. For example, DEGs expressed by canine STS are enriched for genes associated with downregulation of the Friend Leukemia Virus Integration 1 Transcription Factor, FLI1, (Fig 4A). FLI1 acts as a tumor promoter [80, 81], although, interestingly, reduced expression of FLI1 has been shown to induce epithelial-mesenchymal transition in human umbilical vein endothelial cells [82]. Other transcription factors associated with DEGs identified in the present study include FOXP2 and IRX6, neither of which has been well investigated in sarcomas.

Finally, results of the present study may suggest new or improved strategies for targeted treatment of canine soft tissue sarcomas. For example, activation of the proto-oncogenes *MYC*, *ZNF217*, and *WT1* is associated with DEGs expressed by all three tumor types examined in this study (Fig 4), and therapeutic approaches to inhibition of all three in cancers are being developed [83–87]. Our data also suggest that individual tumor types may benefit from tumor type-specific therapeutic approaches. In the present study, FS expressed approximately twice as much KRAS as PNST (S4 Fig). The RAS family of proteins is widely known for their ability to induce cancer, and a drug targeting RAS has recently won FDA approval [88]. Our results also indicate that the Myocyte Enhancer Factor-2 genes MEF2A and MEF2D may play more significant roles in FS than PNST or PWT (Fig 5), and that therapeutic strategies that interfere with MEF2 transcriptional activity [89, 90] may be useful in treating FS. Similarly, the *RELA* proto-oncogene encodes a transcriptionally active subunit of the NF-kB complex that is strongly associated with PNST, but not FS or PWT DEGs (Fig 5) and is being investigated as a therapeutic target [91]. The Ras-related protein gene *RAP1A* was also one of the most significantly expressed genes specific to PWTs (Table 1) and Rap1A promotes cell migration and metastasis [92, 93]. Inhibitors of factors mediating its activation have been examined for use as anti-cancer agents [94].

In summary, results of the present study demonstrate the utility of RNA-seq analysis using archival samples for the identification and analysis of genes expressed by canine soft tissue sarcomas. Our results also identify gene expression patterns that may by valuable in diagnosing and treating these tumors and suggest the involvement of common, tumor-type specific and novel gene regulatory mechanisms in their development.

## Supporting information

S1 Table Sample metadata. (XLSX)

S2 Table Gene expression levels. (XLSX)

S3 Table Numbers of outlier genes per sample. (XLSX)

S4 Table Enrichment of a human soft tissue sarcoma specific gene set for all canine tissue and tumor comparisons. (XLSX)

S5 Table DEGs identified by comparing gene expression among tumors. (XLSX)

S6 Table DEGs identified by comparison of tumors with normal tissues. (XLSX)

S7 Table Statistical values and genes produced by gene set enrichment analysis. (XLSX)

S8 Table Libraries used for gene set enrichment analysis. (XLSX)

S1 Fig Reproducibility of RNA-seq data. Principal component analysis of results obtained from conducting RNA seq for 9 tumors. Reads were obtained in triplicate runs on different days and the results of conducting principal component analysis using the 500 most variable genes are plotted.
(TIF)

S2 Fig Initial unsupervised clustering of tumors. A) Principal component analysis of RNA seq data obtained from 16 formalin fixed paraffin embedded tumors, showing fibrosarcomas (FS, red), peripheral nerve sheath tumors (PNST, green) and perivascular wall tumors (PWT, blue). Tumor type designations were derived from initial histological analysis. B) Unsupervised K-means clustering of data shown in panel A identifies three tumor types, but agreement with histological designations is incomplete.
(TIF)

S3 Fig Normalized expression values for the 50 most significant differentially expressed genes in canine fibrosarcomas. Gene expression differences were calculated between fibrosarcoma samples and combined data from peripheral nerve sheath and perivascular wall tumors. The 25 most significant genes with an increase (A) and decrease (B) in relative expression are shown.
(TIF)

S4 Fig Normalized expression values for the 50 most significant differentially expressed genes in canine peripheral nerve sheath tumors. Gene expression differences were calculated between peripheral nerve sheath tumor samples and combined data from fibrosarcomas and perivascular wall tumors. The 25 most significant genes with an increase (A) and decrease (B) in relative expression are shown.
(TIF)

S5 Fig Normalized expression values for the 50 most significant differentially expressed genes in canine perivascular wall tumors. Gene expression differences were calculated between perivascular wall tumor samples and combined data from fibrosarcomas and peripheral nerve sheath tumors. The 25 most significant genes with an increase (A) and decrease (B) in relative expression are shown.
(TIF)
