# Supplementary figures and images for: Comparative whole transcriptome analysis of gene expression in three canine soft tissue sarcoma types
Source: PLoS One. 2022 Sep 13;17(9):e0273705. doi: 10.1371/journal.pone.0273705 (PMC9469979; doi:10.1371/journal.pone.0273705)

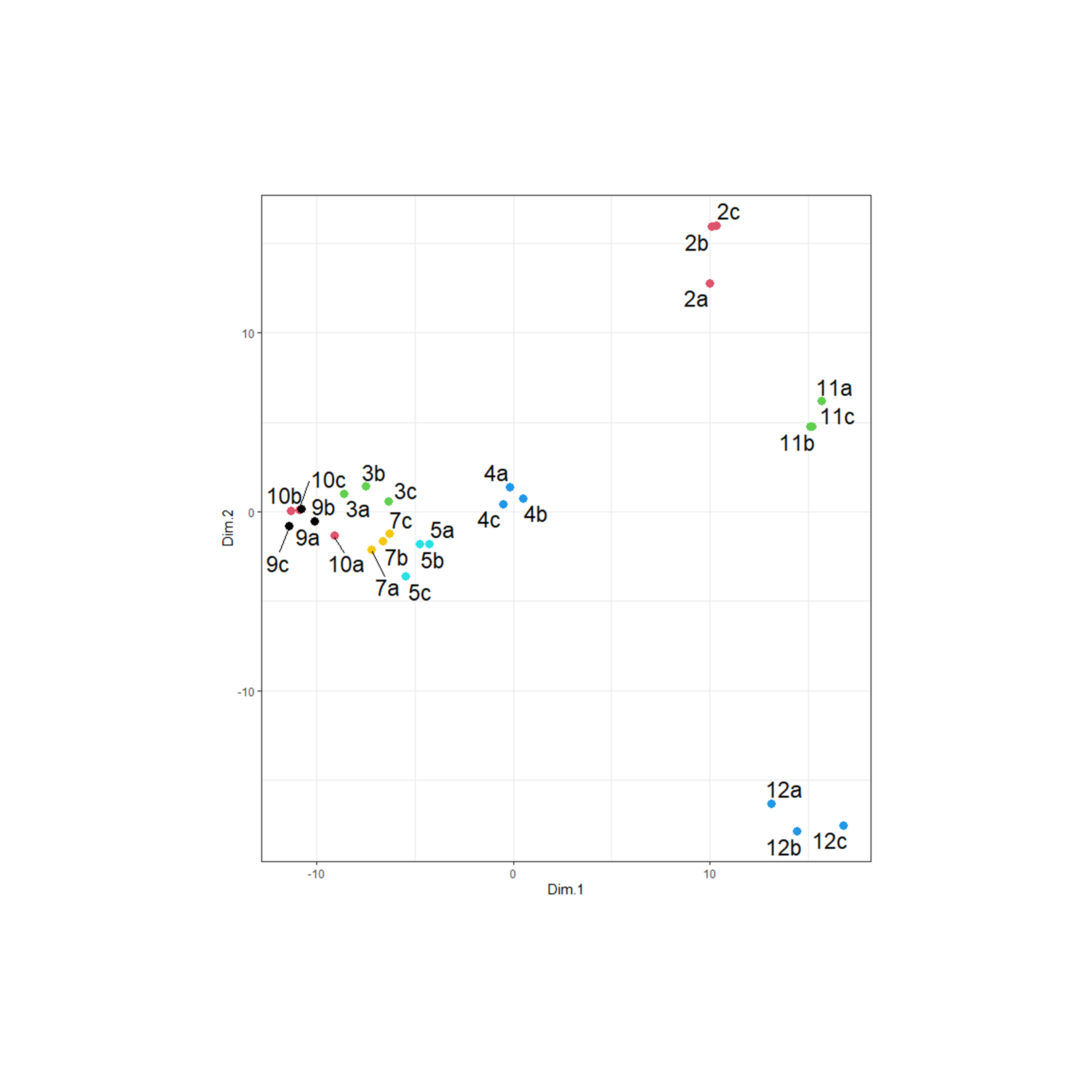

Supplement: S1 Fig — Principal component analysis of results obtained from conducting RNA seq for 9 tumors. Reads were obtained in triplicate runs on different days and the results of conducting principal component analysis using the 500 most variable genes are plotted. (TIF) [file pone.0273705.s009.tif]

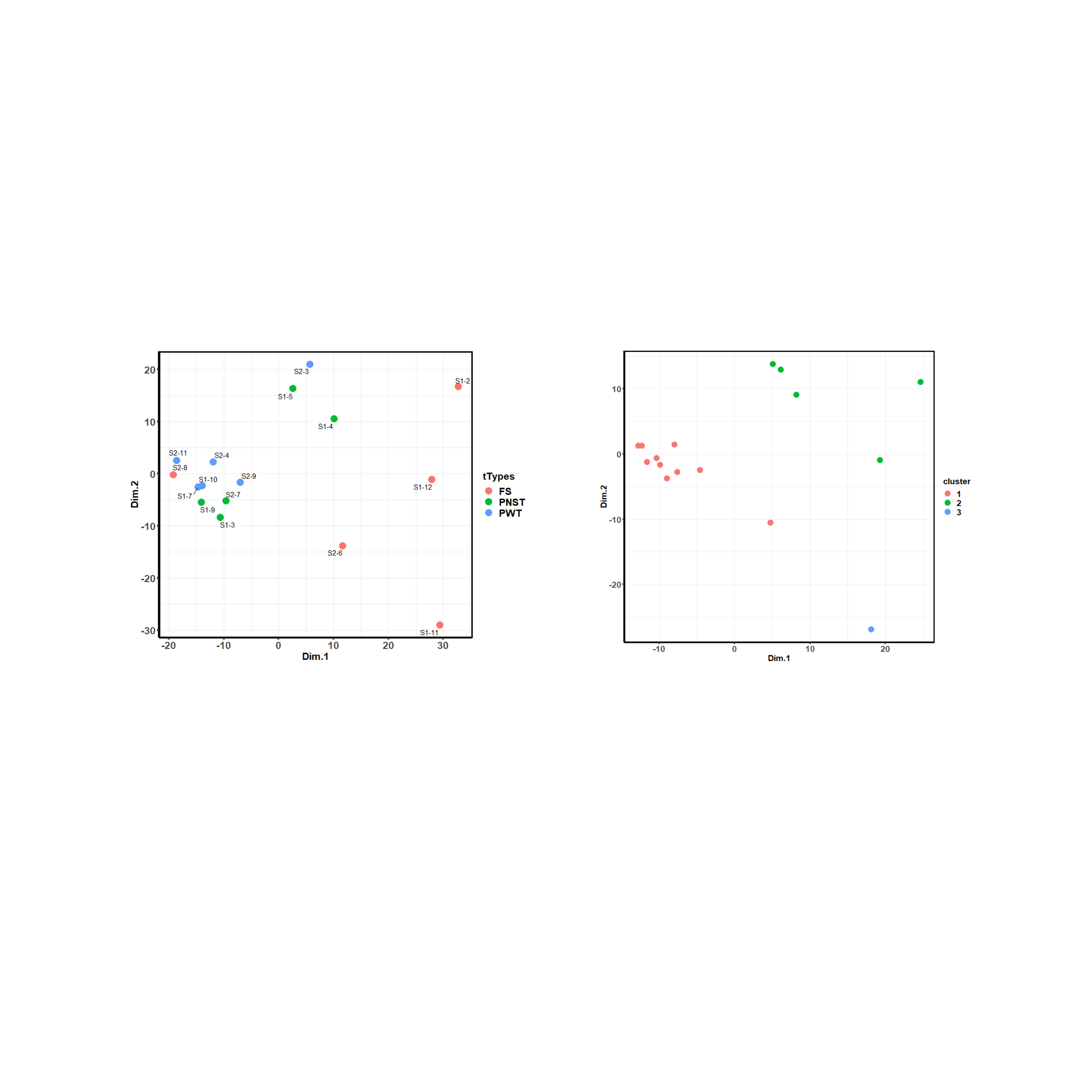

Supplement: S2 Fig — A) Principal component analysis of RNA seq data obtained from 16 formalin fixed paraffin embedded tumors, showing fibrosarcomas (FS, red), peripheral nerve sheath tumors (PNST, green) and perivascular wall tumors (PWT, blue). Tumor type designations were derived from initial histological analysis. B) Unsupervised K-means clustering of data shown in panel A identifies three tumor types, but agreement with histological designations is incomplete. (TIF) [file pone.0273705.s010.tif]

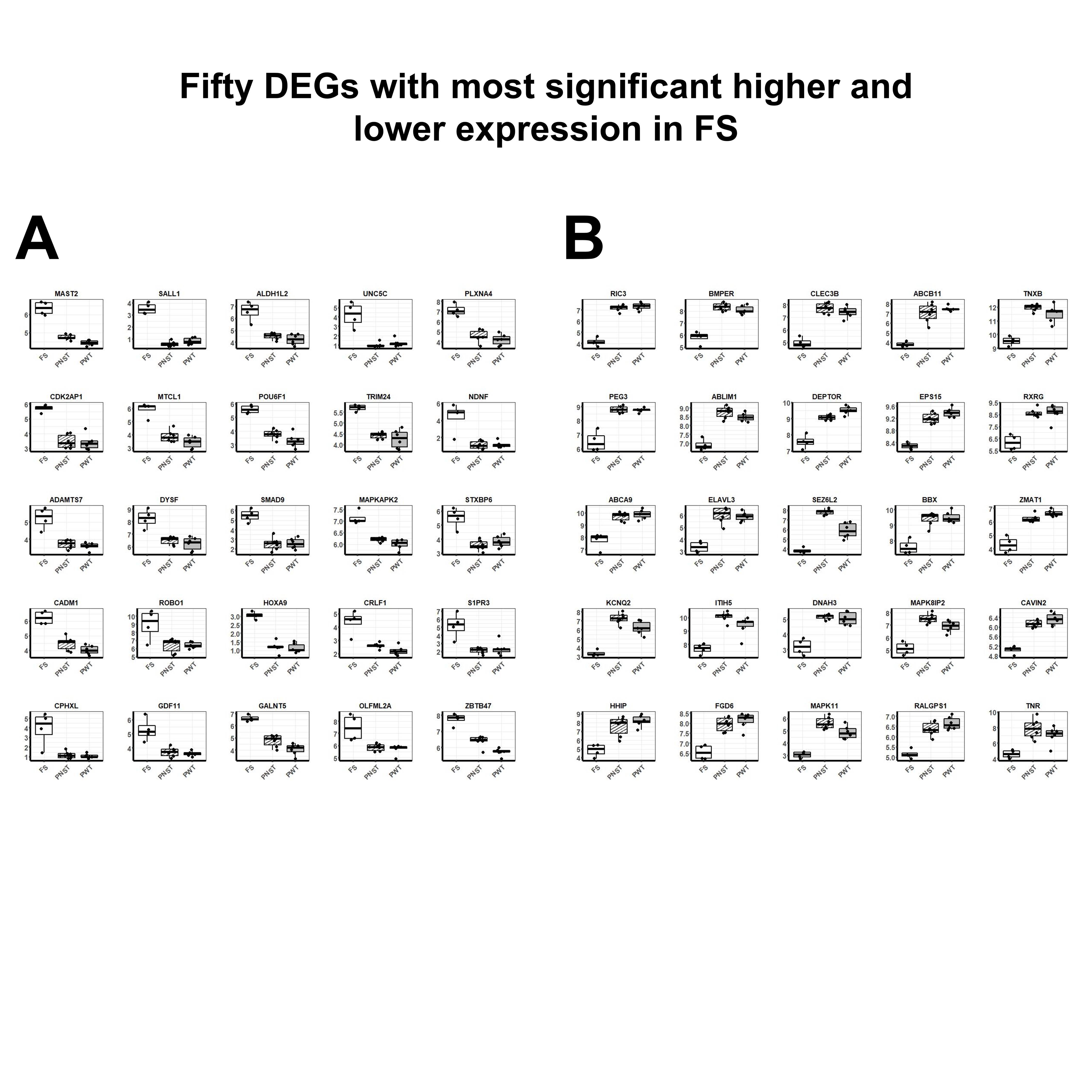

Supplement: S3 Fig — Gene expression differences were calculated between fibrosarcoma samples and combined data from peripheral nerve sheath and perivascular wall tumors. The 25 most significant genes with an increase (A) and decrease (B) in relative expression are shown. (TIF) [file pone.0273705.s011.tif]

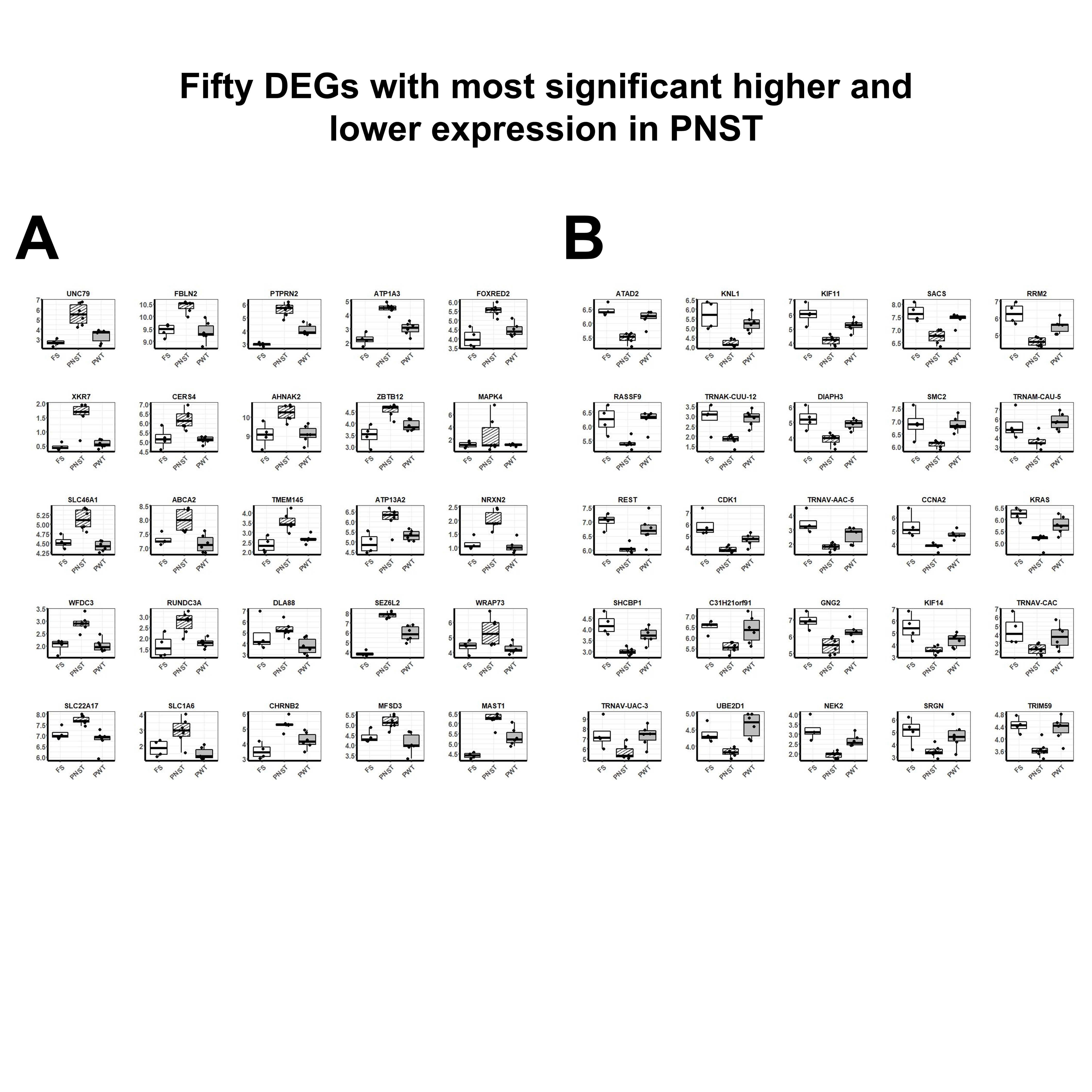

Supplement: S4 Fig — Gene expression differences were calculated between peripheral nerve sheath tumor samples and combined data from fibrosarcomas and perivascular wall tumors. The 25 most significant genes with an increase (A) and decrease (B) in relative expression are shown. (TIF) [file pone.0273705.s012.tif]

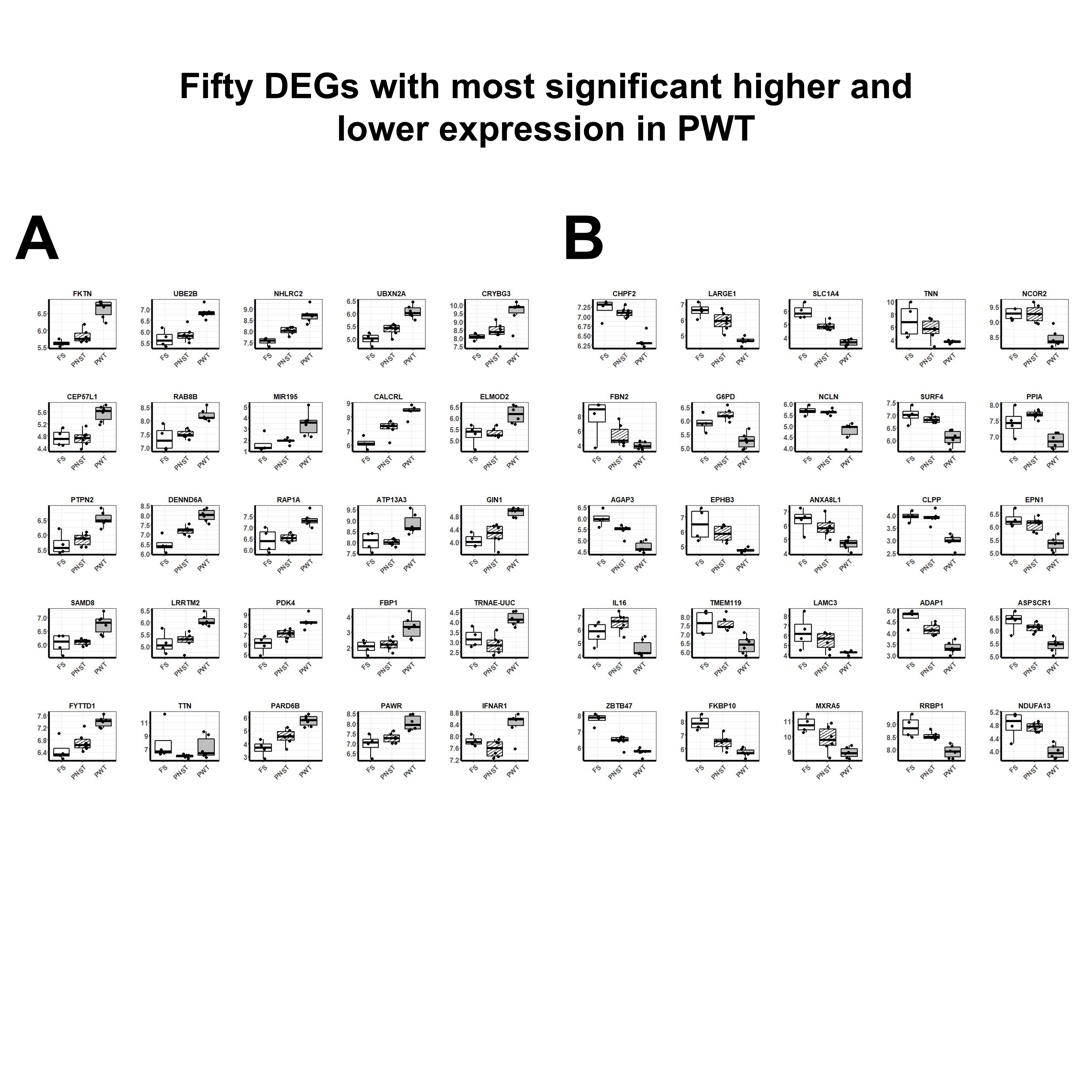

Supplement: S5 Fig — Gene expression differences were calculated between perivascular wall tumor samples and combined data from fibrosarcomas and peripheral nerve sheath tumors. The 25 most significant genes with an increase (A) and decrease (B) in relative expression are shown. (TIF) [file pone.0273705.s013.tif]
